# Use of a Digital Camera to Monitor the Growth and Nitrogen Status of Cotton

**DOI:** 10.1155/2014/602647

**Published:** 2014-02-27

**Authors:** Biao Jia, Haibing He, Fuyu Ma, Ming Diao, Guiying Jiang, Zhong Zheng, Jin Cui, Hua Fan

**Affiliations:** ^1^The Key Laboratory of Oasis Ecological Agriculture, Xinjiang Production and Construction Group/College of Agriculture, Shihezi University, Shihezi, Xinjiang 832000, China; ^2^Xinjiang Shida Sender Technology Co. Ltd, Shihezi, Xinjiang 832000, China

## Abstract

The main objective of this study was to develop a nondestructive method for monitoring cotton growth and N status using a digital camera. Digital images were taken of the cotton canopies between emergence and full bloom. The green and red values were extracted from the digital images and then used to calculate canopy cover. The values of canopy cover were closely correlated with the normalized difference vegetation index and the ratio vegetation index and were measured using a GreenSeeker handheld sensor. Models were calibrated to describe the relationship between canopy cover and three growth properties of the cotton crop (i.e., aboveground total N content, LAI, and aboveground biomass). There were close, exponential relationships between canopy cover and three growth properties. And the relationships for estimating cotton aboveground total N content were most precise, the coefficients of determination (*R* ^2^) value was 0.978, and the root mean square error (RMSE) value was 1.479 g m^−2^. Moreover, the models were validated in three fields of high-yield cotton. The result indicated that the best relationship between canopy cover and aboveground total N content had an *R* ^2^ value of 0.926 and an RMSE value of 1.631 g m^−2^. In conclusion, as a near-ground remote assessment tool, digital cameras have good potential for monitoring cotton growth and N status.

## 1. Introduction

Near-ground remote sensing is playing an increasingly important role in modern agricultural production and precision agriculture [1]. Ground-based observations of crop growth provide fast, real-time, nondestructive, automatic, and relatively inexpensive information about crop status [2]. This information can significantly increase yields by allowing growers to properly time fertilizer application, irrigation, cultivation, harvest, and pest control [3, 4]. Near-ground remote sensing techniques can also be used to quantitatively assess seasonal changes in vegetation growth in order to understand the temporal features of carbon exchange between the atmosphere and the terrestrial biosphere [2, 5].

Many types of instruments have been used for near-ground remote sensing of crop growth and nutrient status during the growing season. Examples of these instruments include GreenSeeker reflectance meters, hyperspectral reflectance meters, Yara N-sensors, and the LAI-2000 canopy analyzer (LI-COR Inc., USA) [6–8]. These devices measure the reflectance or absorption of red and infrared light by either leaves or the entire crop canopy. None of these devices measure canopy properties using green or red reflectance.

Digital cameras have also been used for near-ground remote sensing of crop growth and nutrient status [9–12]. Digital cameras measure red and green reflectance rather than red and infrared reflectance [13]. Digital cameras are consistent, unbiased, and precise. Furthermore, the process of capturing digital images can be automated [14, 15]. Many recent studies have investigated the use of digital cameras to characterize crop growth, nutrient status [4, 16–18], and canopy cover [4, 19–22].

Images taken with digital cameras have been used to establish regression models describing the growth of many major crops including oilseed rape [23], wheat [3, 6, 8, 21, 24, 25], corn [6, 14, 15, 22, 26], and rice [17, 18]. The models are well correlated with crop growth parameters such as leaf area index (LAI), aboveground biomass, and plant N uptake [4, 5, 17–23]. However, few scientists have used images from digital cameras to characterize the growth and N status of cotton under various crop management conditions.

Cotton (*Gossypium hirsutum L*.) is an important crop in the Xinjiang Uyghur Autonomous Region. Cotton growth and yield are affected by the application of fertilizer, especially N fertilizer [27]. The use of near-ground remote sensing to monitor the N status of the cotton crop could improve N use efficiency and then increase cotton yield and quality. The objectives of this study were (i) to develop an image analysis approach for extracting canopy cover with a digital camera, (ii) to calibrate models to estimate aboveground total N content of cotton grown under different N rates, and (iii) to verify the models using data collected from three fields of high-yield cotton.

## 2. Materials and Methods

### 2.1. Experimental Field Sites and Crops

**Figure 1:**
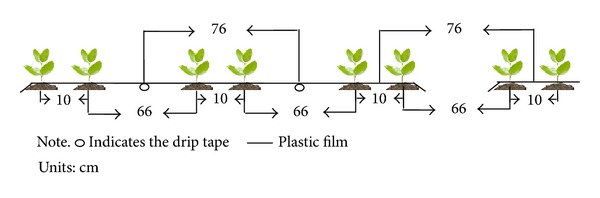
Diagram of row and hill spacing in experiments 1 and 2.

Experiment 1 was conducted at a research site near Shihezi University (44°20′N, 86°3′E) in 2010 and 2011. Selected properties of the heavy loam soil are shown in Table 1. The cotton cultivars were Xinluzao 43 (XLZ 43) and Xinluzao 48 (XLZ 48). The study included five N treatments: 0 kg hm^−2^ (N0), 120 kg hm^−2^ (N1), 240 kg hm^−2^ (N2), 360 kg hm^−2^ (N3), and 480 kg hm^−2^ (N4). Each plot was (6.84 m × 10 m). The plots were arranged in a randomized complete block design with three replicates. Each plot was completely covered by laying three sheets of plastic film (2.02 m wide × 10 m long) edge to edge. Each sheet of plastic film had six rows of cotton, with row spacing of 10 cm, 66 cm, 10 cm, 66 cm, and 10 cm (Figure 1). There was 10 cm between each hill within a row. The row and hill spacing are typical mechanically planted and harvested cotton fields in the region. The plant density was 2.6 × 10^5^ plants hm^−2^. Two drip tapes were laid under each sheet of plastic film in all plots. The emitter discharge rate was 3.2 L h^−1^ and the emitter spacing was 0.30 m. Cotton was sown by hand on April 20, 2010, and on April 18, 2011. On the day after sowing, 300 m^3^ hm^−2^ of water was applied to the plots via drip irrigation to ensure germination. A total of 5400 m^3^ hm^−2^ water was applied during the growing season. The water was divided evenly among 11 irrigation events. Waterproof membrane was buried to a depth of 60 cm around each plot to prevent water movement between plots. All plots were fertilized at planting with 75 kg K_2_O hm^−2^ as potassium chloride, 150 kg P_2_O_5_ hm^−2^ as calcium superphosphate, and 30 kg Zn hm^−2^ as zinc sulfate. Nitrogen fertilizer (urea) was applied via drip irrigation, with 10% of the N applied at planting, 25% applied at budding, 45% applied at blooming, and 20% applied at full boll. The remaining management practices were the same as those used by local farmers.

Experiment 2 was conducted in 2012 at fields belonging to the Agricultural Modernization of Xinjiang Production and Construction Corps (43°06′–45°20′N). Cotton cultivar “Xinluzao 45” (XLZ 45) was grown on Field 9, Company 2, 105th Corp of the 6th Division. Cultivar “Biaoza A_1_” (BZ A_1_) was grown on Field 3, Company 5, 149th Corp of the 8th Division. Cultivar “Shiza 2” (SZ 2) was grown on Field 6, Company 19, 150th Corp of the 8th Division. The total area of each irrigated field was approximately 450 hm^2^. The cotton in all three fields was sown on April 23, 2012. The plant density was 2.5 × 10^5^ plants hm^−2^. Fertilizer was applied at planting at the following rates: 375 kg N hm^−2^ as urea, 150 kg P_2_O_5_ hm^−2^ as calcium superphosphate, and 75 kg K_2_O hm^−2^ as potassium chloride. All other aspects of crop management (i.e., row and hill spacing, irrigation amounts and application times, and fertilizer application times) were the same as in experiment 1.

### 2.2. Instrumentation and Digital Images

Color images of the cotton canopies were captured with a Canon EOS 450D digital camera (Canon Inc., Japan), using a charge-coupled device (CCD) as an image sensor. The following functions were activated on the camera: autofocus, autowhite balance, and automatic exposure time. Autowhite balance refers to the automatic processing of an image by the camera to maintain grey, achromatic, and white colors. The camera was mounted 2.20 m above the ground surface on a monopod that had been designed for this study. The accuracy of images captured with a digital camera is subject to the influence of many factors, including changing weather conditions and illumination intensity [26, 28]. To ensure that the solar angle and light intensity were comparable, the images were all taken on clear days between 12:00 and 14:00 h. For each observation the position of the camera was set so that the edges of the image were midway between cotton rows. Each image contained six full rows of cotton. The image resolution was 4272 × 2848 pixels of 8-bit resolution for red (*R*), green (*G*), and blue (*B*). All images were saved in joint photographic experts group (JPEG) format.

The normalized difference vegetation index (NDVI) and ratio vegetation index (RVI) of the canopies were determined using a GreenSeeker handheld sensor. The values at each site were compared with the canopy cover estimated from the digital images.

### 2.3. Image Segmentation and Calculation of Canopy Cover

The digital images were transferred in JPEG format to a computer and processed to extract red-green-blue (*RGB*) color information. A computer program was developed to extract the *RGB* features of the images and to calculate canopy cover. The program used was Visual Basic version 6.0 for Microsoft Visual Studio.NET platform.

Two methods were used to calculate the cotton canopy cover. In the first method, canopy cover was calculated using the equation developed by Li et al. [4]:

(1) $$\begin{array}{c} \text{CC}=\;\left(1+L\right)\times \left(\frac{\left(G-R\right)}{\left(G+R+L\right)}\right), \end{array}$$

where CC is canopy cover, *G* is canopy reflectance in the green band of the image, *R* is canopy reflectance in the red band of the image, and *L* is the soil baseline with values of 0 for full canopy cover and 1 for bare soil. The value of *L* was set at 0.5 as recommended by Huete [29] and Li et al. [4].

The second method was a modification of the method proposed by Wang et al. [18]. Briefly, a computer algorithm was used to segment cotton plants from the soil background based on the *RGB* color components of each image. The algorithm separated the pixels that comprised each image into one of four classes: sunlit canopy (SC), shaded canopy (ShC), sunlit soil (SS), and shaded soil (ShS).

The details of the algorithm of the computer program are as follows:

 If *R* < 2.6 and *R* < *G* − 5 and *G* > *G* + 5 then canopy pixel
 
* * if *R* + *G* + *B* < 200 then sunlit canopy (SC) pixel
 
* * else shaded canopy (ShC) pixel
 
* * end if
 else soil backgrounds
 
* * if *R* + *G* + *B* > 250 then sunlit soil (SS) pixel
 
* * else shaded soil (ShS) pixel
 
* * end if
 End if

Canopy cover was calculated as the ratio of plant pixels to total pixels in the image. The specific equations were as follows:

(2) $$\begin{array}{c} \text{CC}=P_{\text{SC}}+P_{\text{ShC}},\\ P_{\text{SC}}+P_{\text{ShC}}+P_{\text{SS}}+P_{\text{ShS}}=1, \end{array}$$

where CC is canopy cover, *P*_SC_ is the percentage of pixels in the sunlit canopy, *P*_ShC_ is the percentage of pixels in the shaded canopy, *P*_SS_ is the percentage of pixels in the sunlit soil, and *P*_ShS_ is the percentage of pixels in the shaded soil.

**Figure 2:**
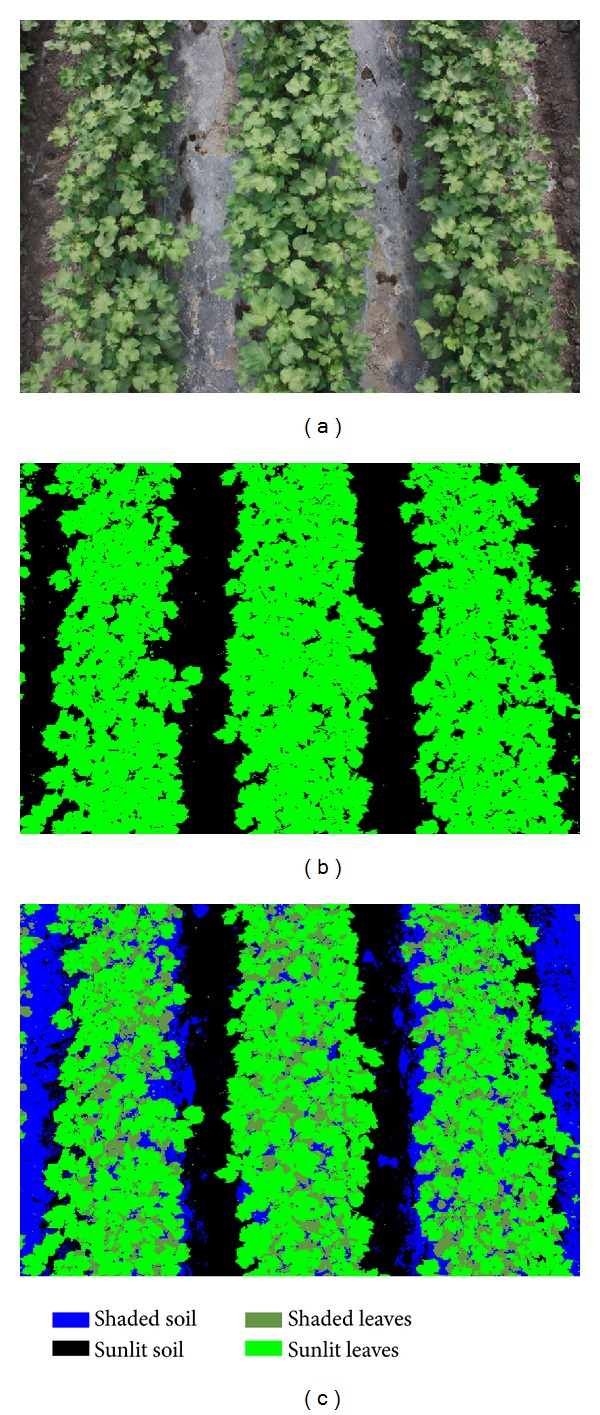
(a) Image of the cotton canopy taken with a digital camera; (b) the proportion of green pixels in the image; (c) the segmentation of the cotton canopy into four parts. The canopy cover in both (b) and (c) was calculated to be 0.6119.

A typical image from the digital camera is shown in Figure 2(a).  Figure 2(b) after it was processed using method 1.  Figure 2(c) shows the image after the pixels were segmented into four groups using method 2. The calculated canopy cover was approximately the same in Figures 2(b) and 2(c).

### 2.4. Cotton Biomass, LAI, and N Content

Cotton plants were destructively sampled on the same day that the images were recorded (Table 2). Leaf area was measured with a LI-3100 C leaf area meter (LI-COR, Lincoln, Nebraska, USA). The LAI (m^2^ m^−2^) was calculated by multiplying the leaf area (m^2^ plant^−1^) by the plant population (plant number m^−2^) in the entire plot or field. Stems, leaves, buds, and bolls were separately dried at 70°C, weighed, and then ground for chemical analysis. The N content of each plant component was using the Kjeldahl method [30]. Total aboveground dry weight (g m^−2^) and total aboveground N content (g m^−2^) were calculated by summing the weight and N content of the individual plant parts.

### 2.5. Statistical Analysis

Correlations between the agronomic parameters and the canopy spectral information were analyzed using SPSS 17.0 software (SPSS v.17.0, SPSS Inc., Chicago, USA). Origin-Pro v.8.5 was used to fit the data and draw the figures. Data from experiment 1 was used to calibrate the regression models for the two cultivars grown with five N rates. The regression models were validated using data from experiment 2, which consisted of three large, representative fields. The performance of the models was generally estimated by comparing the coefficients of determination (*R* ^2^) and the root mean square error (RMSE). The RMSE was calculated using the following equation:

(3) $$\begin{array}{c} \text{RMSE}=\sqrt{\frac{\sum_{n=1}^{n}{\left(O_{m}-S_{m}\right)}^{2}}{n}}, \end{array}$$

where *O*_*m*_ is the observed value, *S*_*m*_ is the simulated values for aboveground total N content, LAI, or aboveground biomass of cotton, and *n* is the number of samples. The precision and accuracy of the model to predict plant growth and N status increased as *R* ^2^ increased and as RMSE declined.

## 3. Results

### 3.1. Relationship of Canopy Cover with NDVI and RVI

**Figure 3:**
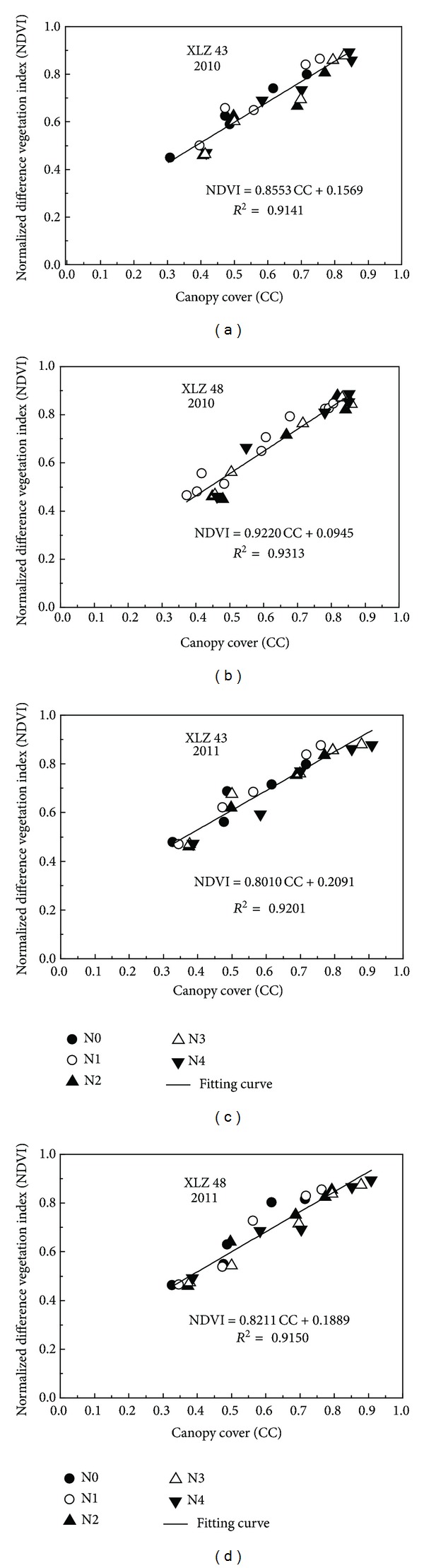
Results from experiment 1 showing the relationship between canopy cover calculated from digital images and normalized difference vegetation index (NDVI) estimated using a GreenSeeker handheld sensor. (a) Cotton cultivar “XLZ 43” in 2010; (b) cotton cultivar “XLZ 48” in 2010; (c) cotton cultivar “XLZ 43” in 2011; (d) cotton cultivar “XLZ 48” in 2011.

**Figure 4:**
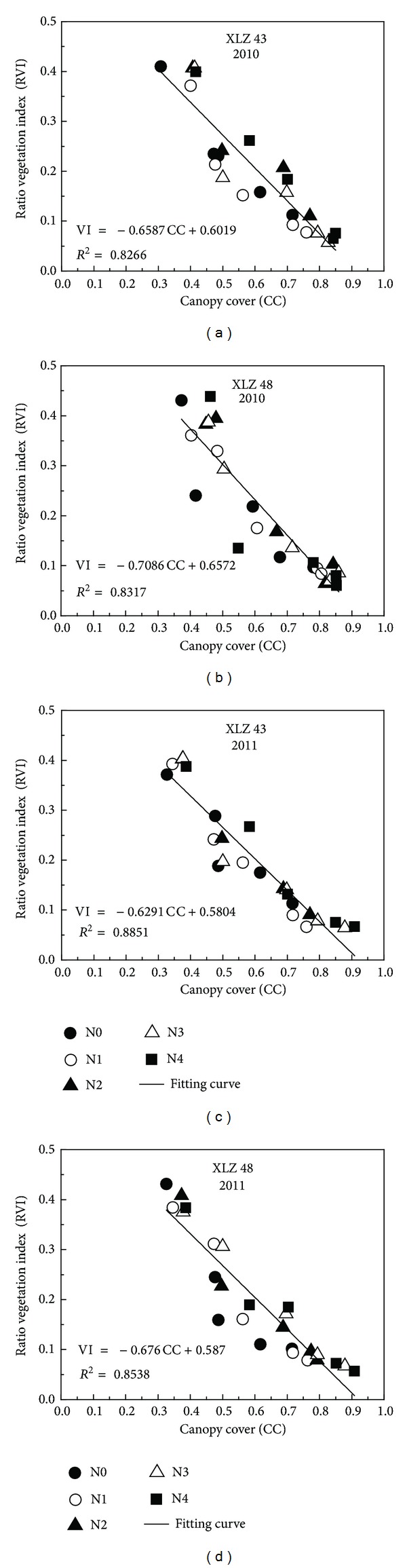
Results from experiment 1 showing the relationship between canopy cover calculated from digital images and ratio vegetation index (RVI) estimated using a GreenSeeker handheld sensor. (a) Cotton cultivar “XLZ 43” in 2010; (b) cotton cultivar “XLZ 48” in 2010; (c) cotton cultivar “XLZ 43” in 2011; (d) cotton cultivar “XLZ 48” in 2011.

The cotton plants were small at emergence. The canopy consisted of only a few, small leaves. Similarly, the values of NDVI and canopy cover were also relatively small. The leaves increased in number and in size as the cotton grew.  Figure 3 shows that NDVI of both cultivars increased as canopy cover increased in 2010 and 2011. Canopy cover and NDVI both reached the maximum at full bloom. In contrast, RVI of both cultivars decreased as estimated canopy cover increased (Figure 4).

The correlation coefficients between canopy cover and the spectral vegetation indices (NDVI and RVI) are shown in Figures 3 and 4. Good relationships were observed between the parameters in both years. Canopy cover from emergence to full bloom was linearly correlated with both NDVI and RVI.

Regression analysis showed significant positive correlation between canopy cover and NDVI (Figure 3). In comparison, there was significant negative linear correlation between canopy cover and RVI (Figure 4). Comparison of the *R* ^2^ values indicated that canopy cover was correlated more closely with NDVI (*R* ^2^ > 0.91, *P* < 0.01) than with RVI (*R* ^2^ > 0.82, *P* < 0.01).

### 3.2. Relationships between Canopy Cover and the Three Crop Properties

In experiment 1, two cotton cultivars were grown with five N rates. Data were fitted using Origin-Pro v.8.5 software. The relationships of canopy cover with three crop properties were best described by the following exponential function:

(4) $$\begin{array}{c} y=ke^{b\times \text{CC}}, \end{array}$$

where *y* is LAI, aboveground biomass, or aboveground total N content, *k* is the initial value of the curve function, and *b* is the curve shaping parameter. The fitted parameters in Table 3 are based on cotton cultivar. The fitted parameters in Table 4 are based on different N rates.

**Figure 5:**
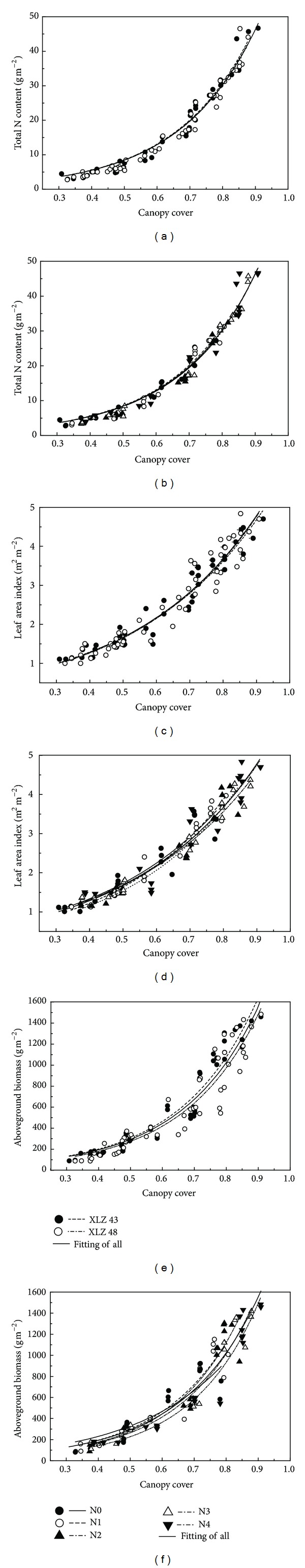
Calibration models describing the relationship between canopy cover and aboveground total N content, LAI, or aboveground biomass in experiment 1. The curves in (a), (c), and (e) were fitted by cotton cultivar: “XLZ 43” or “XLZ 48.” The curves in (b), (d), and (f) were fitted by N rates: 0 kg hm^−2^ (N0), 120 kg hm^−2^ (N1), 240 kg hm^−2^ (N2), 360 kg hm^−2^ (N3), and 480 kg hm^−2^ (N4).

The shapes of the fitted curves describing the relationships between canopy cover and each crop properties were similar for both cultivars (Figures 5(a), 5(c), and 5(e)). Furthermore, there was little variation in the values of either *k* or *b* between the cultivars (Table 3). There was no evidence that cultivar or developmental stage affected the relationships in either year. One reason is that the two cultivars were both upland cotton hybrids. They both had moderate growth habit, with similar leaf shape and mean leaf angle.

There were significant differences in the nonlinear equations describing the relationship of canopy cover with aboveground total N content, LAI, or aboveground biomass among the five N treatments (Figures 5(b), 5(d), and 5(f)). The values of *k* and *b* generally increased as N fertilizer increased (Table 4). The *k* values of the equations ranged from 0.497 to 0.801 for aboveground total N content, from 0.368 to 0.481 for LAI, and from 20.043 to 28.736 for aboveground biomass. The *b* values varied from 4.238 to 4.265 for total aboveground N content, from 2.596 to 2.988 for LAI, and from 3.465 to 4.697 for aboveground biomass. Therefore, under this broad range of conditions, canopy cover was closely related to aboveground total N content, LAI, and aboveground biomass from emergence to full bloom (Tables 3 and 4).

### 3.3. Calibration of the Exponential Models

The exponential models described above were fitted using the calculated canopy cover and the measured aboveground total N content, LAI, or aboveground biomass (Table 5). The exponential equations extracted two variables (*k* and *b*) that appeared to be sufficiently precise for estimating cotton growth and aboveground total N content (Table 5). Figures 5(a)–5(f) and Table 5 show that, from emergence to full bloom, aboveground total N content was more sensitive, with respect to canopy cover (RMSE = 1.479 g m^−2^, *R* ^2^ = 0.978), than was LAI (RMSE = 0.287 m^2^ m^−2^, *R* ^2^ = 0.935) or aboveground biomass (RMSE = 134.412 g m^−2^, *R* ^2^ = 0.901). These results imply that canopy cover values extracted from digital images could be used to estimate the properties of cotton canopy. The white balance and the shutter speeds differed among the images. However, the relationships between canopy cover and the three crop properties were close (Figures 5(a)–5(f)). We conclude that the calibrations were robust across a range of light conditions

### 3.4. Verification Models

**Figure 6:**
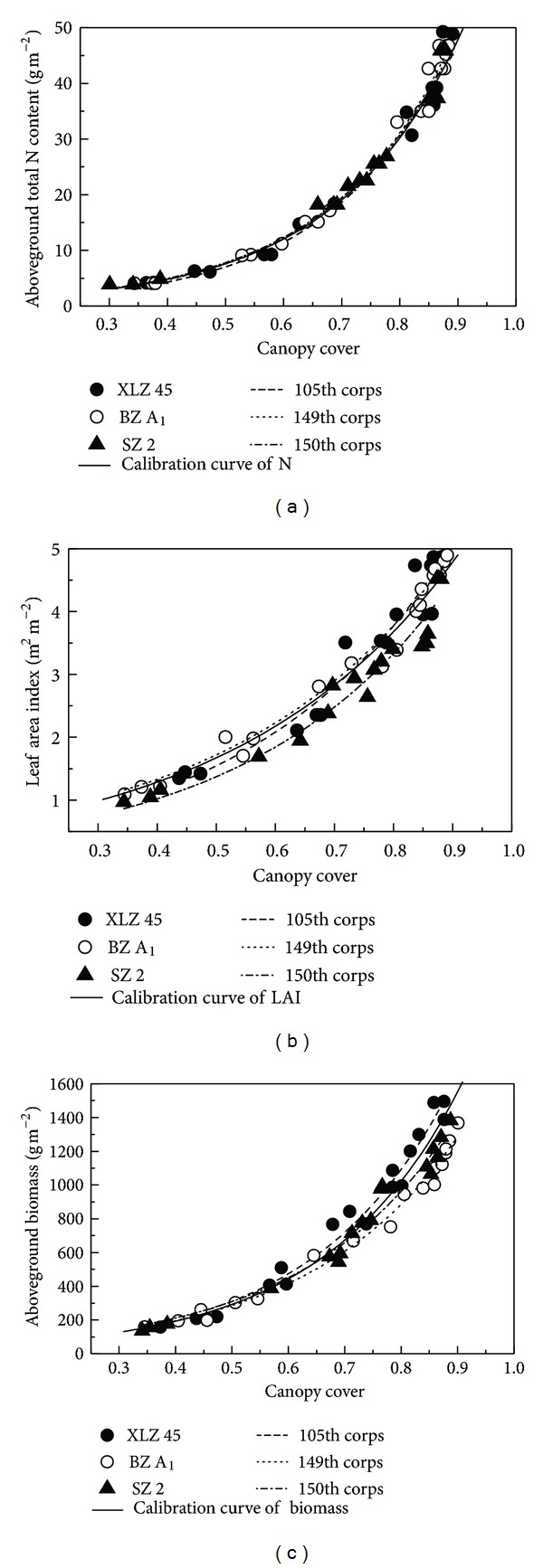
Validation of (4) using the fitted parameters in Table 5 to predict aboveground total N content, LAI, or aboveground biomass in three cotton fields in experiment 2.

The canopy cover equations from experiment 1 were used to estimate the cotton canopy properties. The models were then verified in experiment 2 using data collected from three representative fields belonging to the Agricultural Modernization of Xinjiang Production and Construction Corps. The exponential functions accurately estimated the three crop properties, especially between emergence and full bloom (Figure 6 and Table 5). The *R* ^2^ values were 0.926 for aboveground total N content, 0.842 for LAI, and 0.827 for aboveground biomass. The RMSE values were 1.631 g m^−2^ for aboveground total N content, 0.675 m^2^ m^−2^ for LAI, and 170.156 g m^−2^ for aboveground biomass. These data indicate that canopy cover can be used to accurately estimate the N status of the cotton crop.

## 4. Discussion

Field surveys are the most common method used for determining crop canopy cover. However, field surveys can be time consuming, difficult, and resource consuming. Field surveys may also introduce man-made errors [21, 31]. Remote sensing methods are another way to quantify canopy cover over a large scale [32]. Near-ground remote sensing instruments have been used to monitor crop growth status and estimate yield. For example, NDVI and RVI values measured with a GreenSeeker handheld sensor were highly correlated with crop N status, LAI, and aboveground biomass [32]. However, data collected with some instruments can be difficult to interpret. Furthermore, coarse spatial resolution limits the accuracy and usefulness of most data collected with near-ground remote sensing instruments.

Digital cameras offer several advantages as an alternative tool for near-ground remote sensing of crop canopies. Digital cameras are flexible and cost effective. Images collected with digital cameras have high spatial resolution. Image collection is intuitive, rapid, and nondestructive [2, 15]. Images can be collected over a period of time and then compared to assess temporal changes in crop canopies. Images can be segmented quickly and accurately and then easily archived for future reference [4, 17, 18]. Many researchers have studied the practical use of digital camera images for monitoring crop growth, canopy cover, and/or bare soil measuring [2, 4, 13, 16–20, 25, 33].

In this study, we extracted the *G* and *R* values of the green and red bands from images of cotton canopies (Figures 2(a)–2(c)). Canopy cover was calculated by dividing the number of plant canopy pixels by the total number of pixels in the image (Figures 2(b) and 2(c)). Using this data we were able to accurately estimate canopy cover from emergence to full bloom. This method is simple and easy to apply using images obtained from digital cameras. It is the most common approach for characterizing ground cover at the subpixel scale [18]. Information obtained from the images of digital cameras is more accurate and more useful than information obtained using other instruments because the intact images contain two parts: plant canopy (sunlit canopy and shaded canopy) and soil (sunlit soil and shaded soil) [18]. This method is applicable for extracting canopy cover data of most green plants.

When the canopy coverage of a crop is quite high, the canopy cover value extracted from digital images is often slightly lower than the actual value [18]. This is mainly because the upper part of the canopy shades the lower part of the canopy, thus making it darker in the image. This shaded area, which makes up a relatively small part of the canopy cover, is treated as soil background by computer software and deleted. As a result, the extracted canopy cover slightly underestimates actual canopy cover. However, our results indicated that the underestimation was not large and it does not limit the usefulness of canopy cover for predicting plant growth or N status.

Canopy cover in our study was significantly positively correlated with aboveground total N content, LAI, and aboveground biomass; therefore, it was chosen as the best parameter for determining crop N status [4, 16, 18]. The relationships between canopy cover and aboveground total N content, LAI, and aboveground biomass plant growth were nonlinear and best represented by exponential functions (Figure 5). Moreover, the values of the curve function (*k*) were statistically different among the five N rates (Table 4). This is important because cotton leaves become droopier at high N fertilizer treatment and this increases canopy cover. However, the use of canopy cover to assess N status of crop has limitations. Theoretically, canopy cover ranges from 0 to 1. Actually, the canopy cover is less than 1 when the cotton canopy cover approaches the maximum. At this stage, canopy cover in the image also no longer increases. Once the canopy gets saturated, canopy cover is no longer accurate in reflecting the plant growth conditions [4, 17, 18]. Therefore, assessment of N status using canopy cover can only be done before the cotton canopy reaches the maximum.

The decision coefficient (*R* ^2^) and RMSE of the exponential functions were used to calibrate and verify the models for predicting cotton growth and N status (Table 5). Canopy cover was a good indicator of all the three crop properties in this study. This agrees with previous studies about oilseed rape, wheat, and rice [4, 17, 18, 23]. The highest *R* ^2^ values and the lowest RMSE values in our study were obtained when canopy cover was used in the exponential functions to verify aboveground total N content in different cultivars (Figure 6 and Table 5). This means that, among the three crop properties, canopy cover best estimated aboveground total N.

Nitrogen fertilizer requirement is a function of soil N supply and crop N demand [24, 34]. Traditionally, soil samples have been collected before sowing and analyzed to determine the potential of a soil supply N to the crop. Determination of fertilizer application based on a one-time soil test could result in an economic loss and environmental damage due to either overfertilization or underfertilization. The better approach is to monitor crop N status during the growing season, applying N as needed by the crop. The use of a digital camera is a timely and nondestructive tool for near-ground remote sensing and monitoring. Our results indicate that crop N requirements can be determined using canopy cover. The advantage of using a digital camera combined with a computer with fast software for this application is that the estimate of canopy cover can be combined with other data to estimate the N fertilizer requirement [4].

In the last few years, three-dimensional digital cameras have developed rapidly. If three-dimensional cameras were linked to a decision support system, continuous dynamic information could be obtained about the plant canopy. This information could be used to estimate the crop N fertilizer requirement from year to year. This technology is urgently needed.

## 5. Conclusion

This study demonstrated a reliable, fast, and cost-effective approach for estimating the N status of cotton using digital camera images. High-resolution digital images were acquired in the cotton field, and automated image processing methods were developed to segment the cotton canopy from the soil background. Exponential models for aboveground total N content, LAI, and aboveground biomass showed acceptable precision and accuracy. Canopy cover estimated with images from digital cameras was sufficiently well correlated with aboveground total N content so as to provide useful information about the N fertilizer requirement of the crop. This indicates that digital cameras could be used to nondestructively characterize the growth status of cotton. Overall, our results suggest that near-ground remote sensing technology using digital camera has the potential to improve the efficiency of fertilizer application to agricultural fields, thereby reducing environmental pollution. The use of digital cameras as a tool for near-ground remote sensing in precision agriculture is a new field of research. Future work is needed to improve the methods for image segmentation and image analysis in order to utilize the full potential of this approach.

## Figures and Tables

**Table 1:** Selected soil physical and chemical properties (0–60 cm depth) in experiment 1.

Parameter	Year	
	2010	2011
Clay (%)	21 ± 2.23	19 ± 1.94
Silt (%)	38 ± 2.31	34 ± 1.43
Sand (%)	43 ± 3.19	41 ± 2.26
pH	7.51 ± 0.32	7.72 ± 0.18
Organic matter (mg kg^−1^)	25.46 ± 0.95	26.35 ± 1.11
Alkaline N (mg kg^−1^)	60.83 ± 2.49	58.72 ± 1.65
Olsen P (mg kg^−1^)	28.46 ± 4.24	22.13 ± 1.33
Available K (mg kg^−1^)	342.54 ± 54.13	313.42 ± 32.17
Bulk density (g cm^−3^)	1.26 ± 0.32	1.27 ± 0.19
Saturated volumetric water content (%)	30.11 ± 0.12	32.57 ± 0.25

**Table 2:** Description of experiments for the calibration (experiment 1) and validation (experimentation) of the models.

Year	Location	Cultivar	Sowing date	Treatment	Irrigation	Sample collection
Calibration						
2010	Shihezi University	XLZ 43 XLZ 48	20 April	5 N prates × 3 replications	5400 m^3^ hm^−2^	9 plants per treatment/every twelve days
2011			18 April			

Validation						
2012	105th Corps	XLZ 45	23 April	375 kg N hm^−2^	5400 m^3^ hm^−2^	15 plants per site/every ten days
	149th Corps	BZ A_1_				
	150th Corps	SZ 2				

**Table 3:** Exponential equations showing the relationships between canopy cover and the three crop properties of two cotton cultivars in experiment 1.

Cultivar	Crop property	*k* (mean ± SD)	*b* (mean ± SD)	RMSE	*R* ^2^
XLZ 43	Aboveground total N content	0.712 ± 0.019	4.285 ± 0.020	1.483 g m^−2^	0.966**
	LAI	0.452 ± 0.034	2.622 ± 0.101	0.483 m^2^ m^−2^	0.946**
	Aboveground biomass	37.175 ± 6.092	4.206 ± 0.207	110.714 g m^−2^	0.892*

XLZ 48	Aboveground total N content	0.769 ± 0.017	4.251 ± 0.018	1.437 g m^−2^	0.969**
	LAI	0.438 ± 0.043	2.650 ± 0.128	0.496 m^2^ m^−2^	0.924**
	Aboveground biomass	33.731 ± 8.202	4.208 ± 0.302	146.344 g m^−2^	0.879*

Note: **indicates significant difference at *P* < 0.01;  *indicates significant difference at *P* < 0.05.

**Table 4:** Exponential equations showing the relationships between canopy cover and the three crop properties of two cotton cultivars grown with five N rates in experiment 1.

Crop parameter	N rates	*k* (mean ± S.D.)	*b* (mean ± S.D.)	RMSE	*R* ^2^
Aboveground total N content	N0	0.497 ± 0.034	4.238 ± 0.042	1.793 g m^−2^	0.949**
	N1	0.549 ± 0.021	4.241 ± 0.032	1.459 g m^−2^	0.965**
	N2	0.632 ± 0.027	4.248 ± 0.025	1.328 g m^−2^	0.975**
	N3	0.752 ± 0.024	4.256 ± 0.023	1.287 g m^−2^	0.978**
	N4	0.801 ± 0.037	4.265 ± 0.034	1.384 g m^−2^	0.957**

Leaf area index	N0	0.368 ± 0.079	2.596 ± 0.257	0.565 m^2^ m^−2^	0.864*
	N1	0.384 ± 0.043	2.896 ± 0.157	0.508 m^2^ m^−2^	0.910**
	N2	0.46 ± 0.057	2.907 ± 0.211	0.194 m^2^ m^−2^	0.9618**
	N3	0.465 ± 0.048	2.959 ± 0.129	0.265 m^2^ m^−2^	0.966**
	N4	0.481 ± 0.086	2.988 ± 0.227	0.496 m^2^ m^−2^	0.939**

Aboveground biomass	N0	20.043 ± 11.741	3.465 ± 0.596	348.664 g m^−2^	0.758*
	N1	23.769 ± 10.431	4.378 ± 0.499	225.427 g m^−2^	0.885*
	N2	27.519 ± 14.982	4.632 ± 0.699	139.005 g m^−2^	0.864**
	N3	28.283 ± 8.986	4.674 ± 0.388	124.954 g m^−2^	0.948**
	N4	28.736 ± 6.956	4.697 ± 0.377	192.813 g m^−2^	0.938**

Note: **indicates significant difference at *P* < 0.01; *indicates significant difference at *P* < 0.05.

**Table 5:** Fitted parameters and goodness of fit for calibration equations linking canopy cover in experiment 1 with aboveground total N content, LAI, and aboveground biomass using (1). Validation refers to the goodness of fit for the three equations applied to canopy cover measured at three sites in experiment 2.

	Crop property	Equation	RMSE	*R* ^2^
Calibration	Aboveground total N content	*Y* = 0.619*e* ^4.262×CC^	1.479 g m^−2^	0.978
	Leaf area index	*Y* = 0.448*e* ^2.631×CC^	0.287 m^2^ m^−2^	0.935
	Aboveground biomass	*Y* = 36.297*e* ^4.172×CC^	134.412 g m^−2^	0.901

Validation	Aboveground total N content		1.631 g m^−2^	0.926
	Leaf area index		0.675 m^2^ m^−2^	0.842
	Aboveground biomass		170.156 g m^−2^	0. 827

## Abbreviations

*R*^2^: The coefficients of determination
RMSE: Root mean square error
CC: Canopy cover
LAI: Leaf area index
CCD: Charge-coupled device
NDVI: Normalized difference vegetation index
RVI: Ratio vegetation index
SC: Sunlit canopy
ShC: Shaded canopy
SS: Sunlit soil
ShS: Shaded soil
*R*: Red
*G*: Green
*B*: Blue
JPEG: Joint photographic experts group
*RG**B*: Red-green-blue.
